# Assessment of Screening Approach in Early and Differential Alzheimer’s Disease Diagnosis

**DOI:** 10.3390/antiox10111662

**Published:** 2021-10-22

**Authors:** Laura Ferré-González, Carmen Peña-Bautista, Lourdes Álvarez-Sánchez, Inés Ferrer-Cairols, Miguel Baquero, Consuelo Cháfer-Pericás

**Affiliations:** 1 Alzheimer’s Disease Research Group, Health Research Institute La Fe, 46026 Valencia, Spain; laufegon@alumni.uv.es (L.F.-G.); mariadelcarmen_pena@iislafe.es (C.P.-B.); lourdes_alvarez@iislafe.es (L.Á.-S.); ines_ferrer@iislafe.es (I.F.-C.); baquero_miq@gva.es (M.B.); 2 Division of Neurology, University and Polytechnic Hospital La Fe, 46026 Valencia, Spain

**Keywords:** Alzheimer’s disease, diagnosis model, plasma, biomarker, apolipoprotein E (ApoE), validation, screening

## Abstract

Background: Alzheimer’s disease (AD) is the leading cause of dementia in the elderly population. Currently, diagnosis is based on invasive and expensive techniques, so there is a growing need to look for other possible tests, as well as carry out clinical validation. Studies from the literature showed potential diagnosis models, including some AD risk factors (age, gender, ApoE-ε4 genotype) and other variables (biomarkers levels, neuroimaging). Specifically, a recent model was performed from lipid peroxidation compounds in plasma samples to identify patients with early AD. However, there is a lack of studies about clinical validation of these preliminary diagnosis models. Methods: Plasma samples from participants classified into AD (*n* = 61), non-AD (*n* = 17), and healthy (*n* = 44) were analyzed. In fact, lipid peroxidation compounds were determined by liquid chromatography and mass spectrometry. Then, a previously developed diagnosis model was clinically validated, evaluating some diagnosis indexes. Results: The validation of the preliminary diagnosis model showed satisfactory diagnosis indexes (accuracy 77%, sensitivity 89%, specificity 61%, diagnostic odds ratio 12.5, positive predictive value 76%). Next, a useful screening tool, including the ApoE genotype, was developed, identifying patients with a higher risk of developing AD and improving the corresponding diagnosis indexes (accuracy 82%, sensitivity 81%, specificity 85%, diagnostic odds ratio 23.2, positive predictive value 90.5%). Conclusion: A new screening approach could improve the early, minimally invasive, and differential AD diagnosis in the general population.

## 1. Introduction

Alzheimer’s disease (AD) is the main cause of dementia in the elderly population [1]. More than 50 million people worldwide lived with dementia in 2020, and this number could double by 2040 [2,3]. Actual diagnosis is based on invasive or expensive techniques, such as biomarkers in cerebrospinal fluid (CSF), advanced magnetic resonance imaging (MRI), or positron emission tomography (PET) [4]. In this sense, research has focused on the development of minimally invasive diagnosis models of early AD. However, there is an increasing need to clinically validate these potential models.

Recently developed diagnosis models included some AD risk factors (age, gender, ApoE-ε4 genotype), which showed an evident impact in the trajectory of AD progression [5]. Among them, aging is the most important factor since irreversible processes occur, even in people without pathological characteristics of AD [6,7]. Females showed higher risk of AD, not only because of their longevity compared to men, but also because preclinical studies indicated an increased risk of AD in women during the menopause transition [5,8]. ApoE-ε4 genotype became an important risk factor in recent years because having an ApoE-ε4 allele showed an increased risk; about 60% of the patients diagnosed with AD were ApoE-ε4 carriers [5,9]. Moreover, impaired levels of some CSF or blood metabolites improved the AD diagnosis model [10,11]. The standard CSF AD biomarkers (β-Amyloid-42, t-tau, p-tau) have been assessed in numerous studies, especially for early diagnosis [10,12]. New models based on blood AD biomarkers have been developed, specifically related to β-amyloid pathology and axonal degeneration [13]. Furthermore, blood metabolites related to lipid peroxidation provided a satisfactory diagnosis model to identify early AD patients [14].

In the literature, most of the studies about developing AD diagnosis models used CSF samples [15,16], as well as neuroimaging techniques [17,18,19], since research suggested that measurable changes in proton emission tomography, magnetic resonance imaging, and CSF biomarkers occurred some years before the onset of clinical symptoms. However, these techniques involved disadvantages due to the high cost or the invasiveness of the tests [15]. However, a recently developed model was performed in plasma samples from early AD patients, obtaining satisfactory results [14]. Other developed diagnosis models were mainly based on participants diagnosed with mild cognitive impairment (MCI) or AD [16,17,18,19]. Thus, all of them showed important clinical symptoms.

The aim of this study is to carry out a clinical validation of an AD diagnosis model and to develop a potential screening methodology, improving the early and differential AD diagnosis in the whole population.

## 2. Materials and Methods

### 2.1. Participants

This study was undertaken with 122 participants who were recruited from the Neurology Unit at the University and Polytechnic Hospital La Fe (Valencia (Spain)). The sample comprised 54 men and 68 women between 45 and 78 years old. They were either healthy or had mild cognitive disorder. The study was conducted according to the guidelines of the Declaration of Helsinki and approved by the Ethics Committee at the Health Research Institute La Fe (Valencia) (protocol code 2019/0105, date 22 May 2019). Informed consent was obtained from all subjects involved in the study.

For this study, participants were classified according to standard diagnosis criteria of the National Institute on Aging–Alzheimer’s Association [20], including determination of CSF biomarkers of AD. Furthermore, neuropsychological evaluation based on global state (Clinical Dementia Rating, CDR) [21], mini-mental state examination (MMSE) [22], and neuropsychological assessment (Repeatable Battery for Assessment of Neuropsychological Status, RBANS) [23] was carried out. According to these evaluations, participants were classified into early AD (*n* = 61), non-AD (*n* = 17), and healthy (*n* = 44) groups. The early AD group included participants with positive CSF AD biomarkers (β-amyloid-42 < 725 pg·mL^−1^, total tau (t-tau) > 485 pg·mL^−1^, phosphorylated tau (p-tau) > 56 pg·mL^−1^) and mild cognitive impairment (CDR ≤ 0.5, MMSE ≤ 27, RBANS.DM ≤ 85). The non-AD group included participants with negative CSF AD biomarkers (β-amyloid-42 > 725 pg·mL^−1^, t-tau < 485 pg·mL^−1^, p-tau < 56 pg·mL^−1^) and cognitive impairment in at least one of these tests (CDR ≤ 0.5, MMSE ≤ 27, RBANS.DM ≤ 85). The healthy group included participants with negative levels for CSF AD biomarkers (β-amyloid-42 > 725 pg·mL^−1^, t-tau < 485 pg·mL^−1^, p-tau < 56 pg·mL^−1^) and normal cognitive tests (CDR ≤ 0.5, MMSE ≥ 27, RBANS.DM ≥ 85). Participants with major brain disorders, traumatic brain injuries, and psychiatric disorders were excluded, as well as participants who were not able to complete the neuropsychological evaluations.

### 2.2. Sample Collection and Treatment

Blood samples from participants were collected between 8 and 10 a.m. following the established procedures and clinical guides in AD diagnosis. Samples were centrifuged for 15 min at 1.500 g, and plasma fraction was separated in a new tube and stored at −80 °C until analysis.

The treatment and analysis of the samples were carried out as described by Peña-Bautista et al. [24].

### 2.3. Statistical Analysis

Statistical analysis was performed using IBM Statistical Package for the Social Sciences software version 23.0 (SPSS, Inc., Chicago, IL, USA). Descriptive characteristics were summarized as follows: qualitative variables were expressed as absolute frequencies and percentages (%), and quantitative variables were expressed as medians and inter-quartile range (IQR). In all cases, the statistical significance was set at *p* < 0.05.

For the validation of the preliminary diagnosis model, the equation obtained in the previous study to determine the probability of suffering from preclinical AD status was applied to the new values obtained in the validation cohort:

Pr (preclinical AD) = *e* ^LP^/(1 + *e* ^LP^) where LP = −6.566 − 0.153 × Female + 0.164 × Age—11.622 × A − 28.241 × B − 3.277 × C + 2.457 × D + 6.391 × E + 8.988 × F − 0.174 × G + 0.315 × H + 9.298 × I − 0.323 × J (A: 15-*epi*-15-F2t-IsoP; B: PGE_2_; C: 15-keto-15-E_2t_-IsoP; D: 15-keto-15-F_2t_-IsoP; E: 15-E2t-IsoP; F: PGF_2α_; G: 4(RS)-4-F_4t_-NeuroP; H: 1a,1b-dihomo-PGF_2α_ I: 10-*epi*-10-F_4t_-NeuroP J: 14(RS)-14-F_4t_-NeuroP) [14].

The parameters of accuracy, sensitivity, specificity, diagnostic odds ratio, and positive predictive value (PPV) were calculated from the validation results. Statistical significance between test characteristics was obtained by comparing 95% confidence intervals (CI).

## 3. Results

### 3.1. Patients’ Characteristics

Demographic and clinical characteristics of the participants are described in Table 1. The clinical variables allowed us to differentiate among participants groups. As can be seen, the CSF biomarkers levels (ß-amyloid42, t-tau, p-tau) and the neuropsychological evaluation (CDR, MMSE, RBANS.DM) allowed us to identify AD patients from healthy and non-AD participants. As expected, the healthy and non-AD groups showed higher levels of β-amyloid-42 and lower levels of t-tau and p-tau than the AD group, and the AD and non-AD groups showed some impairment in neuropsychological tests scores.

The analyte concentrations found in plasma samples from participant groups are summarized in Table 2. As observed, most compounds showed higher levels for the healthy group.

### 3.2. Diagnosis Model Validation

The predictor variables used in the previously developed diagnosis model were age, gender, and plasma levels of the lipid peroxidation compounds shown in Table 2 [14]. As can be seen in Table 3, the diagnosis indexes of the model were calculated together with their 95% CI. The sensitivity was approximately 89% and the specificity was 61.4%. The accuracy of the model was 77.4%, the PPV was 76.4%, and the odds ratio was 12.5.

### 3.3. Screening Approach Development for Clinical Practice Application

**Figure 1:**
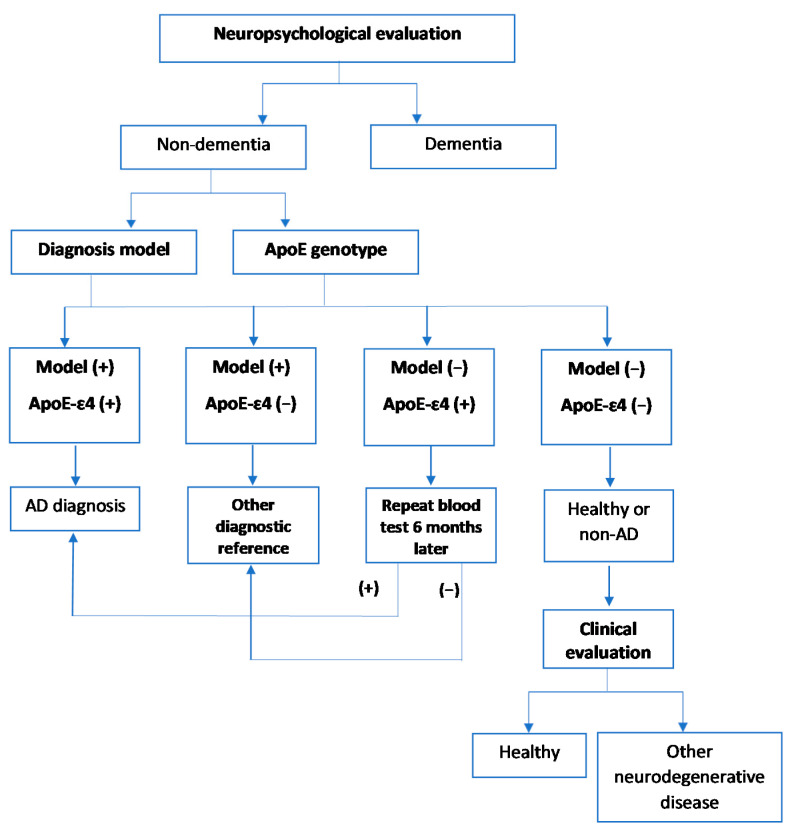
Decision tree as screening approach in early and differential AD diagnosis.

In the development of a potential screening approach, a decision tree was performed considering the neuropsychological tests (dementia, non-dementia), the previously validated diagnosis model (positive, negative), and the genotype ApoE (allele ε4 carrier, non-carrier) (Figure 1).

First, neuropsychological tests were performed to differentiate patients with dementia from those who did not develop dementia. Then, blood samples were taken from patients with non-dementia to determine lipid peroxidation compounds and genotype. According to the blood analysis, (i) if both tests were positive, an AD diagnosis was confirmed; (ii) if the model response was positive and the genotype was negative, other specific tests would be performed on the patient (e.g., CSF analysis); (iii) if the model response was negative and the genotype was positive, a new blood test would be performed 6 months later to re-evaluate the model response; (iv) if both tests were negative, AD diagnosis would be discarded, and a clinical evaluation may be carried out to determine whether these patients suffer from other neurodegenerative pathologies.

In this sense, the decision tree could constitute a useful screening approach to identify patients with AD in the general population, since, in some cases (blood tests +/+ or −/−), expensive or invasive tests would be avoided. Regarding the diagnosis ability of the decision tree in these cases (AD diagnosis or healthy/non-AD), the sensitivity was approximately 81% and the specificity was 84.6%. The accuracy of the model was 82.2%, the PPV was 90.5%, and the odds ratio was 23.2 (Table 4).

## 4. Discussion

The multivariate model developed by Peña-Bautista et al. includes highly significant variables in AD diagnosis, such as age, gender, and 10 biomarkers of lipid peroxidation, so it constitutes a promising diagnostic tool [14]. The present study carried out the corresponding validation and improved its diagnosis capacity by adding the ApoE genotype blood test.

Lipid peroxidation is an important factor in the development of neurodegenerative diseases. Biomarkers of lipid peroxidation in the brain have been linked to histological lesions produced in neurodegenerative diseases, such as β-amyloid plaques [25]. In other words, biomarkers of lipid peroxidation showed a correlation with AD-specific biomarkers in CSF and with neuropsychological status. Previous studies observed an association of AD with altered levels of some lipid peroxidation compounds in plasma, which may be useful in clinical practice to differentiate healthy people from patients with mild cognitive impairment (MCI) due to AD [24,26]. In addition, the analysis of plasma samples would enable future application to the general population as a prevention approach [27].

The model used for the preclinical diagnostics was based on analysis of plasma samples from participants with preclinical AD and healthy elderly participants [14]. This implies that preclinical AD patients showed positive CSF biomarkers (amyloid, t-tau p-tau), but they did not show altered neuropsychology yet. This is an interesting starting point, as many studies are already conducted with AD patients at a more advanced stage [17,28,29]. Therefore, this model would be useful in further prevention programs, since it would detect early AD cases, improving its management and treatment in the future.

From the previous diagnosis model, a decision tree was developed, increasing the diagnostic power. Specifically, it was observed that adding ApoE genotyping (blood sample), although some sensitivity was lost, the other diagnosis indexes improved considerably. In this sense, previous studies in the literature agree that the ApoE ε4 allele is an important genetic risk factor that is linked to both sporadic and hereditary AD [30,31]. Moreover, recent evidence suggests that ε4 carrier status may confer the highest risk in women aged 65–75 years [32]. As such, ApoE ε4 carrier status may provide a very specific and distinct approach to the development of AD prevention strategies, as lifestyle, genomics, AD comorbidities, and other biological and behavioral factors may be affected by the presence of the ε4 allele [33]. Another study provided evidence that sex and the presence of ApoE ε4 allele were associated with CSF levels of inflammatory biomarkers [34]. Consequently, it may be a marker of the neurodegenerative process in the course of AD [35].

It should be noted that the present study developed a complete minimally invasive screening approach, while some previous studies about diagnostic models of AD using non-invasive tests (urine, blood) required invasive or expensive tests (CSF biomarkers, PET) to confirm preliminary results [28,36]. In addition, the developed decision tree differentiated between AD and other neurodegenerative diseases. This is an important advantage since most of the previously published works used neuroimaging techniques [37,38] or neuropsychology to differentiate between the different stages of AD or between different types of dementias [39,40], but they did not use blood biomarkers tests to complete the decision.

Regarding limitations, the present study carried out a clinical validation of the previous diagnosis model and the developed decision tree using a small sample size. However, early AD participants were defined from CSF biomarkers with high accuracy.

## 5. Conclusions

The growing need to identify patients with early AD propitiated the clinical validation of a potential diagnosis model, including some AD risk factors (age, gender, ApoE-ε4 genotype) and other variables (lipid peroxidation biomarkers). This minimally invasive diagnosis model showed satisfactory diagnostic indexes for a differential AD diagnosis. In addition, the incorporation of the ApoE genotype in the developed decision tree constituted a promising tool for general population screening, which would reduce costs for the public health system. Nevertheless, further work to validate the complete screening approach is required as a previous step in clinical practice and in the development of further prevention programs.

## Figures and Tables

**Table 1:** Participants’ demographic and clinical variables.

Variable		Healthy (*n* = 44)	Non-AD (*n* = 17)	AD (*n* = 61)
*Demographic characteristics*				
Age (years, median (IQR))		62 (55–68)	65 (61–69)	70 (66–74)
Gender (female *n* (%))		26 (59.1%)	7 (41.2%)	35 (57.4%)
Level of education *n* (%)	Basic Secondary University	14 (31.8%) 9 (20.5%) 21 (47.7%)	10 (58.8%) 4 (23.5%) 3 (17.6%)	31 (50.8%) 13 (21.3%) 17 (27.9%)
*Clinical characteristics*				
ApoE (ε4 carrier *n* (%))		7 (15.9%)	5 (29.4%)	40 (65.6%)
β-Amyloid42 (pg·mL^−1^, median (IQR))		1044 (875–1421)	947 (804–1136)	568 (469–665)
t-tau (pg·mL^−1^, median (IQR))		214 (174–283)	244 (180–299)	556 (424–751.50)
p-tau (pg·mL^−1^, median (IQR))		34 (24–42)	40 (27–58.50)	92 (68.50–110)
CDR (median (IQR))		0 (0–0.5)	0.5 (0–0.5)	0.5 (0.5–0.5)
MMSE (median (IQR))		29 (28–29)	27 (22.50–28.50)	25 (21–27)
RBANS.DM (median (IQR))		98 (94–102)	78 (58–84)	56 (40–71.50)

ApoE: apolipoprotein E; CDR: Clinical Dementia Rating; MMSE: mini-mental state examination; RBANS: Repeatable Battery for the Assessment of Neuropsychological Status (DM: delayed memory).

**Table 2:** Plasma levels of lipid peroxidation compounds.

Variable (nmol·L^−1^)	Healthy Group (*n* = 44) Median (1st, 3rd Quartile)	Non-AD Group (*n* = 17) Median (1st, 3rd Quartile)	AD Group (*n* = 61) Median (1st, 3rd Quartile)
15(R)-15-F_2t_-IsoP	0.48 (0.23–0.68)	0.43 (0.18–0.60)	0.33 (0.23–0.61)
PGE_2_	0.28 (0.16–0.35)	0 (0–0.10)	0 (0–0.25)
15-keto-15-E_2t_-IsoP	0.76 (0.01–1.17)	0 (0–0.14)	0 (0–0.21)
15-keto-15-F_2t_-IsoP	0.48 (0.18–0.82)	0.23 (0.04–0.31)	0.20 (0.03–0.34)
15-E_2t_-IsoP	0.91 (0.60–1.36)	0.20 (0–0.28)	0.28 (0–0.70)
PGF_2α_	0.36 (0.23–0.73)	0 (0–0.78)	0.60 (0–0.78)
4(*RS*)-4-F_4t_-NeuroP	3.16 (1.18–4.38)	0.87 (0–1.06)	1.10 (0–1.65)
1a,1b-dihomo-PGF_2α_	3.03 (0–4.25)	0 (0–0.61)	0 (0–0)
10-epi-10-F_4t_-NeuroP	0.15 (0.01–0.24)	0 (0–0.20)	0.08 (0–0.20)
14(*RS*)-14-F_4t_-NeuroP	1.25 (0.54–2.05)	0 (0–0.68)	0.25 (0–0.98)

**Table 3:** Prediction effect of the model.

Parameter	(95% CI)
Sensitivity (%)	88.7 (78.5–94.4)
Specificity (%)	61.4 (46.6–74.3)
Accuracy (%)	77.4 (68.5–84.3)
PPV (%)	76.4 (65.4–84.7)
Odds ratio	12.5 (4.6–33.7)

PPV: Positive Predictive Value.

**Table 4:** Prediction effect of the model and genotype.

Parameter	(95% CI)
Sensitivity (%)	80.9 (67.5–89.6)
Specificity (%)	84.6 (66.5–93.9)
Accuracy (%)	82.2 (71.9–89.3)
PPV (%)	90.5 (77.9–96.2)
Odds ratio	23.2 (6.4–84.3)

## Data Availability

The data presented in this study are available on request from m.consuelo.chafer@uv.es (C.C.-P.). The data are not publicly available due to individuals’ data protection.

## References

[1] Durmugier J., Sabia S. (2020). Epidemiology of Alzheimer’s disease: Latest trends. Rev. Prat..

[2] Nichols E., Szoeke C.E.I., Vollset S.E., Abbasi N., Abd-Allah F., Abdela J., Aichour M.T.E., Akinyemi R.O., Alahdab F., Asgedom S.W. (2019). Global, regional, and national burden of Alzheimer’s disease and other dementias, 1990–2016: A systematic analysis for the Global Burden of Disease Study 2016. Lancet Neurol..

[3] Prince M., Wimo A., Guerchet M., Ali G.-C., Wu Y.-T., Prina M. (2015). World Alzheimer Report 2015: The Global Impact of Dementia. Alzheimer’s Dis. Int..

[4] Hane F.T., Robinson M., Lee B.Y., Bai O., Leonenko Z., Albert M.S. (2017). Recent Progress in Alzheimer’s Disease Research, Part 3: Diagnosis and Treatment. J. Alzheimer’s Dis..

[5] Riedel B.C., Thompson P.M., Brinton R.D. (2016). Age, APOE and sex: Triad of risk of Alzheimer’s disease. J. Steroid Biochem. Mol. Biol..

[6] Breijyeh Z., Karaman R. (2020). Comprehensive Review on Alzheimer’s Disease: Causes and Treatment. Molecules.

[7] Sengoku R. (2020). Aging and Alzheimer’s disease pathology. Neuropathology.

[8] Scheyer O., Rahman A., Hristov H., Berkowitz C., Isaacson R.S., Diaz Brinton R., Mosconi L. (2018). Female Sex and Alzheimer’s Risk: The Menopause Connection. J. Prev. Alzheimer’s Dis..

[9] Armstrong R.A. (2019). Risk factors for Alzheimer’s disease. Folia Neuropathol..

[10] Olsson B., Lautner R., Andreasson U., Öhrfelt A., Portelius E., Bjerke M., Hölttä M., Rosén C., Olsson C., Strobel G. (2016). CSF and blood biomarkers for the diagnosis of Alzheimer’s disease: A systematic review and meta-analysis. Lancet Neurol..

[11] Varma V.R., Oommen A.M., Varma S., Casanova R., An Y., Andrews R.M., O’Brien R., Pletnikova O., Troncoso J.C., Toledo J. (2018). Brain and blood metabolite signatures of pathology and progression in Alzheimer disease: A targeted metabolomics study. PLoS Med..

[12] Howell J.C., Watts K.D., Parker M.W., Wu J., Kollhoff A., Wingo T.S., Dorbin C.D., Qiu D., Hu W.T. (2017). Race modifies the relationship between cognition and Alzheimer’s disease cerebrospinal fluid biomarkers. Alzheimers. Res. Ther..

[13] Lashley T., Schott J.M., Weston P., Murray C.E., Wellington H., Keshavan A., Foti S.C., Foiani M., Toombs J., Rohrer J.D. (2018). Molecular biomarkers of Alzheimer’s disease: Progress and prospects. Dis. Models Mech..

[14] Peña-Bautista C., Álvarez-Sánchez L., Ferrer I., López-Nogueroles M., Cañada-Martínez A.J., Oger C., Galano J.-M., Durand T., Baquero M., Cháfer-Pericás C. (2021). Lipid Peroxidation Assessment in Preclinical Alzheimer Disease Diagnosis. Antioxidants.

[15] Ritchie C., Smailagic N., Ladds E.C., Noel-Storr A.H., Ukoumunne O., Martin S., Ritchie C. (2013). CSF tau and the CSF tau/ABeta ratio for the diagnosis of Alzheimer’s disease dementia and other dementias in people with mild cognitive impairment (MCI). Cochrane Database of Systematic Reviews.

[16] Meyer P., Savard M., Poirier J., Morgan D., Breitner J. (2019). Hypothesis: Cerebrospinal fluid protein markers suggest a pathway toward symptomatic resilience to AD pathology. Alzheimer’s Dement..

[17] Vemuri P., Gunter J.L., Senjem M.L., Whitwell J.L., Kantarci K., Knopman D.S., Boeve B.F., Petersen R.C., Jack C.R. (2008). Alzheimer’s disease diagnosis in individual subjects using structural MR images: Validation studies. Neuroimage.

[18] Aggarwal N., Rana B., Agrawal R., Kumaran S. (2015). A combination of dual-tree discrete wavelet transform and minimum redundancy maximum relevance method for diagnosis of Alzheimer’s disease. Int. J. Bioinform. Res. Appl..

[19] Qiu S., Joshi P.S., Miller M.I., Xue C., Zhou X., Karjadi C., Chang G.H., Joshi A.S., Dwyer B., Zhu S. (2020). Development and validation of an interpretable deep learning framework for Alzheimer’s disease classification. Brain.

[20] Albert M.S., DeKosky S.T., Dickson D., Dubois B., Feldman H.H., Fox N.C., Gamst A., Holtzman D.M., Jagust W.J., Petersen R.C. (2011). The diagnosis of mild cognitive impairment due to Alzheimer’s disease: Recommendations from the National Institute on Aging-Alzheimer’s Association workgroups on diagnostic guidelines for Alzheimer’s disease. Alzheimer’s Dement..

[21] Hughes C.P., Berg L., Danziger W., Coben L.A., Martin R.L. (1982). A New Clinical Scale for the Staging of Dementia. Br. J. Psychiatry.

[22] Folstein M.F., Folstein S.E., McHugh P.R. (1975). “Mini-mental state”. J. Psychiatr. Res..

[23] Randolph C., Tierney M.C., Mohr E., Chase T.N. (1998). The Repeatable Battery for the Assessment of Neuropsychological Status (RBANS): Preliminary Clinical Validity. J. Clin. Exp. Neuropsychol..

[24] Peña-Bautista C., Vigor C., Galano J.M., Oger C., Durand T., Ferrer I., Cuevas A., López-Cuevas R., Baquero M., López-Nogueroles M. (2018). Plasma lipid peroxidation biomarkers for early and non-invasive Alzheimer Disease detection. Free Radic. Biol. Med..

[25] Montagne A., Zhao Z., Zlokovic B.V. (2017). Alzheimer’s disease: A matter of blood–brain barrier dysfunction?. J. Exp. Med..

[26] Bradley-Whitman M.A., Lovell M.A. (2015). Biomarkers of lipid peroxidation in Alzheimer disease (AD): An update. Arch. Toxicol..

[27] García-Blanco A., Baquero M., Vento M., Gil E., Bataller L., Cháfer-Pericás C. (2017). Potential oxidative stress biomarkers of mild cognitive impairment due to Alzheimer disease. J. Neurol. Sci..

[28] Pan X., Fei G., Lu J., Jin L., Pan S., Chen Z., Wang C., Sang S., Liu H., Hu W. (2016). Measurement of Blood Thiamine Metabolites for Alzheimer’s Disease Diagnosis. EBioMedicine.

[29] Wang N., Chen J., Xiao H., Wu L., Jiang H., Zhou Y. (2019). Application of artificial neural network model in diagnosis of Alzheimer’s disease. BMC Neurol..

[30] Serrano-Pozo A., Das S., Hyman B.T. (2021). APOE and Alzheimer’s disease: Advances in genetics, pathophysiology, and therapeutic approaches. Lancet Neurol..

[31] Poirier J., Miron J., Picard C., Gormley P., Théroux L., Breitner J., Dea D. (2014). Apolipoprotein E and lipid homeostasis in the etiology and treatment of sporadic Alzheimer’s disease. Neurobiol. Aging.

[32] Neu S.C., Pa J., Kukull W., Beekly D., Kuzma A., Gangadharan P., Wang L.-S., Romero K., Arneric S.P., Redolfi A. (2017). Apolipoprotein E Genotype and Sex Risk Factors for Alzheimer Disease. JAMA Neurol..

[33] Berkowitz C.L., Mosconi L., Rahman A., Scheyer O., Hristov H., Isaacson R.S. (2018). Clinical Application of ApoE in Alzheimer’s prevention: A precision medicine approach. J. Prev. Alzheimer’s Dis..

[34] Duarte-Guterman P., Albert A.Y., Inkster A.M., Barha C.K., Galea L.A.M. (2020). Inflammation in Alzheimer’s Disease: Do Sex and APOE Matter?. J. Alzheimer’s Dis..

[35] Prendecki M., Florczak-Wyspianska J., Kowalska M., Ilkowski J., Grzelak T., Bialas K., Kozubski W., Dorszewska J. (2019). APOE genetic variants and apoE, miR-107 and miR-650 levels in Alzheimer’s disease. Folia Neuropathol..

[36] Janelidze S., Mattsson N., Palmqvist S., Smith R., Beach T.G., Serrano G.E., Chai X., Proctor N.K., Eichenlaub U., Zetterberg H. (2020). Plasma P-tau181 in Alzheimer’s disease: Relationship to other biomarkers, differential diagnosis, neuropathology and longitudinal progression to Alzheimer’s dementia. Nat. Med..

[37] Sharma N., Kolekar M.H., Jha K. (2020). Iterative Filtering Decomposition Based Early Dementia Diagnosis Using EEG with Cognitive Tests. IEEE Trans. Neural Syst. Rehabil. Eng..

[38] Miltiadous A., Tzimourta K.D., Giannakeas N., Tsipouras M.G., Afrantou T., Ioannidis P., Tzallas A.T. (2021). Alzheimer’s Disease and Frontotemporal Dementia: A Robust Classification Method of EEG Signals and a Comparison of Validation Methods. Diagnostics.

[39] Jin M., Deng W. (2018). Predication of different stages of Alzheimer’s disease using neighborhood component analysis and ensemble decision tree. J. Neurosci. Methods.

[40] Ziso B., Larner A.J. (2019). Codex (Cognitive Disorders Examination) Decision Tree Modified for the Detection of Dementia and MCI. Diagnostics.

